# Total Hip Arthroplasty Combined with Proximal Femoral Reconstruction Effectively Treats Severe Hip Deformities: A Novel Osteotomy Technique

**DOI:** 10.1111/os.14136

**Published:** 2024-06-18

**Authors:** Xianyue Shen, Rongwei Zhang, Jiawei Mei, Xianzuo Zhang, Wei Huang, Chen Zhu

**Affiliations:** ^1^ Department of Orthopedics, Centre for Leading Medicine and Advanced Technologies of IHM, The First Affiliated Hospital of USTC, Division of Life Sciences and Medicine University of Science and Technology of China Hefei China

**Keywords:** Osteotomy, Proximal femoral deformities, Proximal femoral reconstruction, Total hip arthroplasty

## Abstract

**Objective:**

Total hip arthroplasty (THA) combined with proximal femoral reconstruction is a novel osteotomy technique developed to address severe hip deformities. There is a paucity of robust clinical and radiological evidence regarding the outcomes of this novel osteotomy technique. This study aimed to evaluate the clinical and radiological outcomes of THA combined with proximal femoral reconstruction during the early follow‐up.

**Methods:**

This is a retrospective case series of 63 hips who underwent THA combined with proximal femoral reconstruction at a single institution between January 2020 and July 2023. The mean age of patients was 39.6 ± 12.6 years. The mean follow‐up was 25.6 ± 3.8 months. Surgical characteristics and perioperative variables were evaluated to assess the efficacy of this technique. Harris hip score (HHS) was utilized to evaluate hip function. Leg length discrepancy (LLD) was evaluated in X‐ray. The incidence of major adverse events including deep vein thrombosis (DVT), osteolysis, nonunion of the osteotomy, intraoperative femoral fracture, and infection was also evaluated. Paired‐samples *t*‐test was used to compare preoperative and postoperative HHS and LLD.

**Results:**

The mean operative time was 125.1 min. The mean size of the acetabular components used was 45.2 mm, and the stem size was 7.5. The primary friction interface was ceramic‐on‐ceramic, accounting for 92.1% of cases. The average length of hospital stay was 8.5 days. The mean cost of treatment was 46,296.0 Yuan. There was a significant improvement in postoperative HHS (*p* < 0.001) and LLD (*p* < 0.001) compared to preoperative values. The incidence of deep venous thrombosis was 4.8%; osteolysis rates for the cup and stem were 4.8% and 6.4%, respectively. The non‐union and dislocation rates were 1.6% and 3.2%, respectively. There was no incidence of postoperative infection.

**Conclusion:**

The novel osteotomy surgical procedure yields reliable and impressive clinical and radiological outcomes, with minimal complications. We advocate for its use in complex primary THA cases involving severe proximal femoral deformities.

## Graphical abstract

Total hip arthroplasty (THA) combined with proximal femoral reconstruction is a novel osteotomy technique developed to address severe hip deformities yields reliable and impressive clinical and radiological outcomes, with minimal complications. We advocate for its use in complex primary total hip arthroplasty cases involving severe proximal femoral deformities.

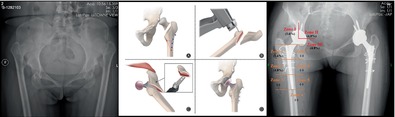

## Introduction

Total hip arthroplasty (THA) is a commonly performed orthopedic procedure to reconstruct the hip joint function by replacing a damaged hip with an artificial one. It is often referred to as the “the operation of the century.”^1^ However, in some complex cases, hip pathology may be accompanied by deformities or defects at the proximal femur, rendering traditional THA treatment methods inadequate to meet the patient's needs.^2^

The treatment of severe anatomical deformity of the hip joint has always been an important cause of confusion for orthopedic surgeons and is associated with reduced patient satisfaction.^3^, ^4^ For severely deformed hip joints, achieving and maintaining femoral head reconstruction to a true acetabulum is challenging. If the cup is placed in a non‐anatomical position, it may result in a high rate of dislocation, persistent lameness, and component loosening. For example, in patients with severe developmental dysplasia of the hip (DDH), significant bone morphological abnormalities, narrowing of the femoral medullary cavity, excessive anterior tilt of the femur, and associated soft tissue contractures and leg length discrepancies often require additional procedures to ensure a successful outcome.^5^ Procedures such as trochanteric osteotomy and subtrochanteric osteotomy have been widely used to address these challenges,^6^, ^7^, ^8^ but these procedures have a steep learning curve. In addition, they often necessitate the use of modular femoral stems, which increases the risk of complications such as wear, corrosion, and fracture at the modular junction. Moreover, these prostheses can be costly. Especially for the Chinese population with a narrow femoral medullary cavity, modular prostheses (S‐ROM) may not be the ideal choice.^9^

In this context, to address the challenges posed by severe anatomical variations in the hip joint, a novel femoral proximal reconstruction technique during THA has been developed. This technique was first reported by our team in 2019 and involves a long oblique osteotomy extending from the proximal border of the lesser trochanter to the lateral aspect of the femur.^10^ This approach simplifies femoral shortening osteotomy, uses a larger osteotomy block to facilitate fixation and osseointegration, artificially increases the medullary canal diameter, and reduces hospitalization costs. The aim of this study was to evaluate the clinical effectiveness of THA combined with femoral proximal reconstruction during the perioperative period and early follow‐up, as well as to assess the incidence of adverse events.

## Patients and Methods

### Patients

This was a retrospective study of adult patients who underwent THA with a proximal femoral osteotomy at our hospital between January 1, 2020 and July 1, 2023. Ethical approval for this study was granted by the institutional review board, Ethics Committee of the First Affiliated Hospital of USTC (No.2023‐RE‐358). The study was performed in accordance with the Declaration of Helsinki.^11^ All participants provided written informed consent prior to enrollment. All surgical procedures were performed by two surgeons who have undergone fellowship in joint arthroplasty and who perform over 500 arthroplasties annually.

The inclusion criteria were: (1) age ≥ 18 years; (2) patients who underwent total hip arthroplasty due to hip pathologies, with proximal femoral osteotomy performed during the operation, (3) availability of follow‐up and radiographic data. Patients who had undergone proximal femoral osteotomy with plate fixation, those who were lost to follow‐up, and those with incomplete records and imaging were excluded. Patient characteristics, clinical details and radiological images were obtained from the patient notes.

### Surgical Procedure

**FIGURE 1:**
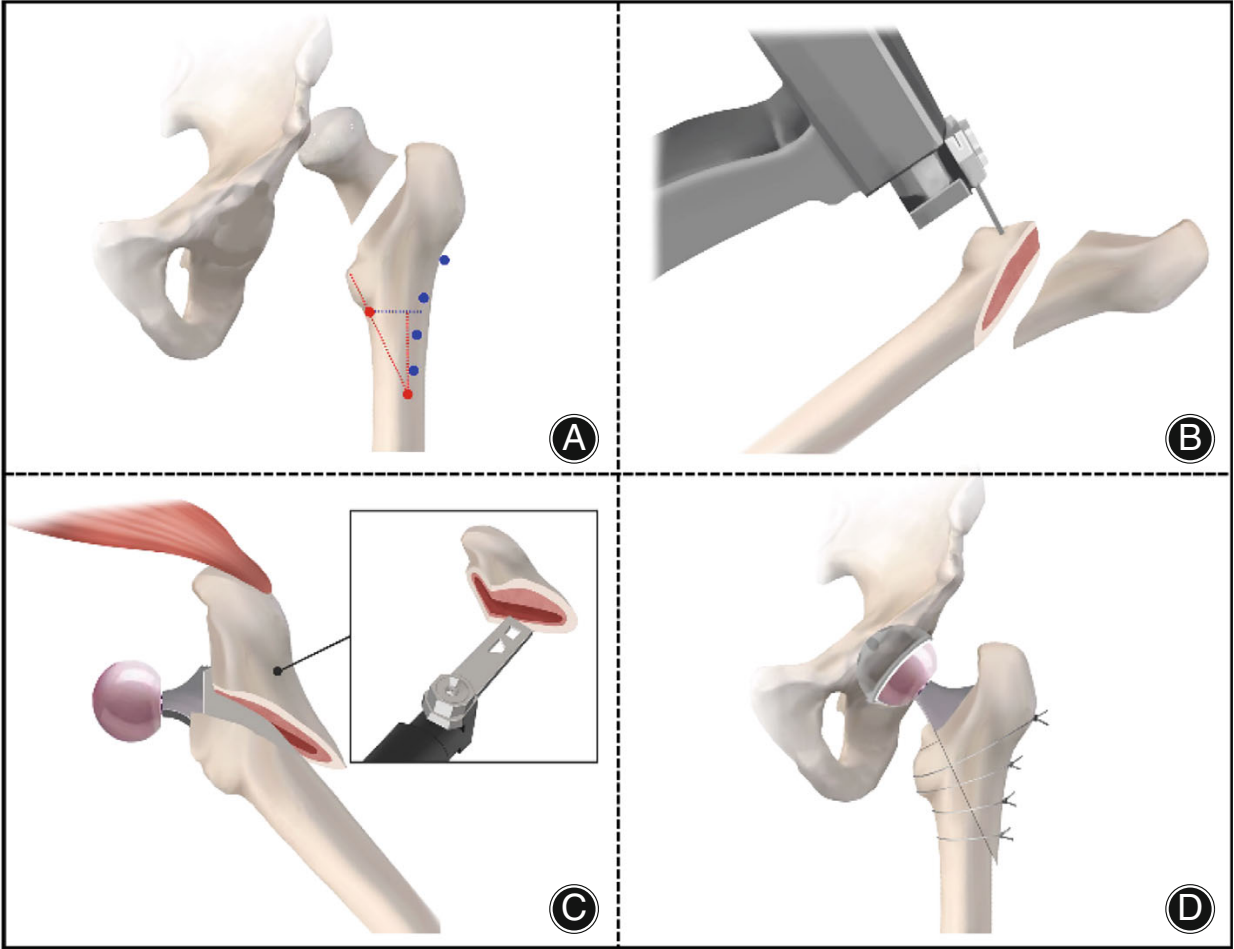
Surgical schematic diagram of total hip arthroplasty combined with proximal femoral reconstruction. (A) After removing the femoral head, the route of proximal femoral osteotomy is determined based on preoperative planning. (B) The femur is appropriately resected to adjust the limb length of the affected side. (C) The lager trochanteric osteotomy bone flap is trimmed to match the proximal shape of the stem, facilitating the implantation of the stem. (D) Several steel wires are used to bind the lager trochanteric osteotomy bone flap, stem, and femur together, and reconstruct the hip joint.

All patients received prophylactic intravenous antibiotics 2 h prior to surgery and 2 g of tranexamic acid intravenously 30 min before skin incision. The surgical approach was selected based on the surgeon's preference, primarily involving the posterolateral and direct anterior approaches. All surgeries were performed under general anesthesia. The external rotator muscles were routinely exposed up to the piriformis, transected proximally, and reflected to expose and incise the posterior capsule. A vertical femoral neck osteotomy was performed above the lesser trochanter, and the femoral head was removed. An oblique osteotomy was performed above the lesser trochanter on the proximal femur to obtain a mobilizable greater trochanteric bone flap, with the attached gluteus medius and minimus muscles. The distal femur was manually reamed to implant an appropriately sized stem, and the osteotomy surface of the mobilizable greater trochanteric bone flap was contoured into a groove to match the stem. The tension of the gluteus medius was adjusted by manipulating the greater trochanteric bone flap, which was then secured with several loops of steel wire. For highly dislocated hip joints, necessary distal femoral osteotomy was performed to facilitate joint reduction and limb length balance. In cases with extremely narrow femoral medullary canals, a small groove was created on the lateral side of the femur and pre‐circled with steel wire before manual reaming to assist in controlling the direction of bone splitting (Figure 1). The decision to insert a drain and the reconstruction and closure of the lateral muscle group in layers were then made intraoperatively.

### Postoperative Management

Postoperatively, an immediate accelerated rehabilitation plan, implemented by a multidisciplinary team, was initiated.^12^ Rehabilitation exercises, including ankle pump exercises, isometric quadriceps contractions, and moderate active hip flexion and extension exercises, commenced immediately after surgery. On the first postoperative day, the patient was instructed to walk without weight‐bearing. Crutches were used to assist partial weight‐bearing 1 week after surgery, and patients gradually transitioned to walking without crutches after 6 weeks. Low‐molecular‐weight heparin was administered 12 h postoperatively to prevent deep vein thrombosis (DVT), and flurbiprofen axetil injection was administered for analgesia. Hip anteroposterior X‐ray and weight‐bearing full‐length X‐rays of the lower extremity were reviewed 24 h postoperatively, and a duplex ultrasound of the lower limb veins was performed within 48 h postoperatively.

### Clinical and Radiological Evaluations

Details of the surgical procedures, operation time, prosthesis size, and length of stay for the patients were obtained from the medical records. All patients underwent clinical and radiological evaluations preoperatively and at the final follow‐up. The hip function was assessed using the Harris hip score (HHS) preoperatively and at the final follow‐up. The incidence of major adverse events (DVT, infection, dislocation, and non‐union) during follow‐up was assessed. Implant‐bone interfaces in each zone were analyzed in X‐ray at the final follow‐up, according to the methods described by Gruen *et al*.^13^ and DeLee and Charnley *et al*.^14^ In addition, correction of the pelvic tilt and LLD were also investigated by a rehabilitation physician in the clinic. Leg length discrepancy was evaluated in X‐ray using Sayed‐Noor *et al*.'s method.^15^

### Statistical Analysis

Continuous variables are expressed as mean ± standard deviation (SD), while categorical variables are summarized as frequency (percentage). Paired‐samples *t*‐test was used to compare preoperative and postoperative variables. *p* values of < 0.05 were considered indicative of statistical significance. Statistical analysis was performed using SPSS for Windows (version 22.0; IBM, Chicago, IL, USA).

## Results

### Demographic and Clinical Characteristics

The study retrospectively evaluated the clinical and radiological data of 43 (eight males, 35 females) patients (63 hips) who underwent THA combined with proximal femoral reconstruction (Details of the cases are provided in Table S1). The mean age of patients in this series was 39.6 years. The main indication for surgery was DDH (88.9%), followed by pyogenic hip arthritis (3.2%). According to Crowe classification,^16^ 52 cases (92.9%) were classified as Crowe type IV, three cases (5.4%) as Crowe type III, and one case (1.8%) as Crowe type I. The mean follow‐up was 25.6 months (range, 19–37). The demographic and clinical characteristics are summarized in Table 1.

**TABLE 1:** Demographic and clinical information of patients.

Variables	Value
Number of patients (hips)	43 (63 hips)
Body mass index (kg/m^2^)	21.6 ± 3.2
Age (years)	39.6 ± 12.5
Sex	
Male	8 (18.6%)
Female	35 (81.4%)
Laterality	
Left	35 (55.6%)
Right	28 (44.4%)
Diagnosis	
Developmental dysplasia of the hip	56 (88.9%)
Pyogenic hip arthritis	2 (3.2%)
Juvenile idiopathic osteoarthritis	1 (1.6%)
Other deformities	4 (6.3%)

### Perioperative Variables and Clinical Outcomes

**FIGURE 2:**
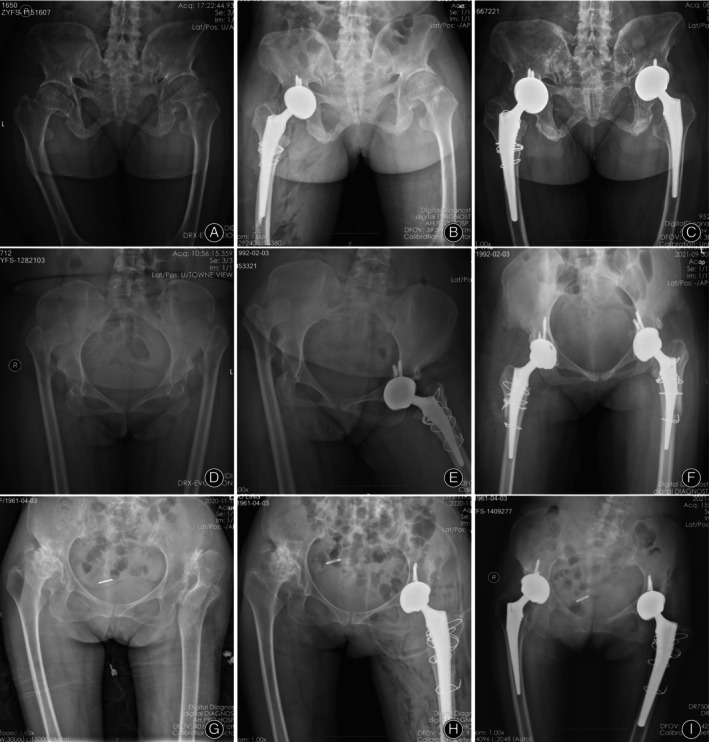
Representative cases presentation. (A–C) Case 1, female, bilateral proximal femur deformity, total hip arthroplasty combined with proximal femoral reconstruction performed on the right hip. (A) Preoperatively, (B) postoperatively, and (C) at 3‐year follow‐up. (D–F) Case 2, female, bilateral DDH (Crowe‐IV), total hip arthroplasty combined with proximal femoral reconstruction performed on both hips. (D) preoperatively, (E) at 1‐year follow‐up (left), and (F) at 2‐year follow‐up (left). (G–I) Case 3, female, right proximal femur deformity. total hip arthroplasty combined with proximal femoral reconstruction performed on the left hip. (G) Preoperatively, (H) postoperatively, and (I) at 2‐year follow‐up.

The perioperative variables of the included patients were collected. The mean operative time for THA combined with proximal femoral reconstruction in the 63 surgeries was 125.1 ± 39.5 min. The average diameter of the acetabular components used was 45.2 ± 2.7 mm, while the average size of the femoral stem was 7.5 ± 1.4. The primary friction interface between the acetabulum and femoral head was ceramic‐on‐ceramic, accounting for 92.1%. The average length of stay (LOS) in this series was 8.5 days and the average hospitalization cost was 46296.0 ± 12942.4 Yuan. The mean HHS at the last follow‐up (89.6 ± 3.2) was significantly better than the mean preoperative HHS (44.8 ± 4.5) indicating significant improvement in hip function (*p* < 0.001). Analysis of radiographic measurements showed that the postoperative LLD was significantly decreased compared to preoperative LLD (*p* < 0.001) (Table 2). Figure 2 shows a set of representative cases.

**TABLE 2:** Summary of surgical characteristics and perioperative variables.

Variables	Value
Operation time (minutes)	125.1 ± 39.5
Follow‐up time (months)	25.6 ± 3.8
Friction interface	
CoC	58
CoP	4
MoP	1
Acetabular component size (mm)	45.2 ± 2.7
Femoral stem size^a^	7.5 ± 1.4
Length of stay (days)	8.5 ± 3.5
Hospitalization expenses (yuan)	46296.0 ± 12942.4
Harris score^b^	
Preoperative	44.8 ± 4.5
Last follow‐up	89.6 ± 3.2
Leg‐length discrepancy (cm)^b^	
Preoperative	4.1 ± 0.9
Last follow‐up	1.3 ± 0.6

Abbreviations: CoC, ceramic‐on‐ceramic; CoP, ceramic‐on‐polyethylene; MoP, metal‐on‐polyethylene.

^a^ Among the included cases, 5 cases used Wagner Cone (Zimmer, Warsaw, USA) stem.

^b^ Represents *p* < 0.001.

### Complications

**FIGURE 3:**
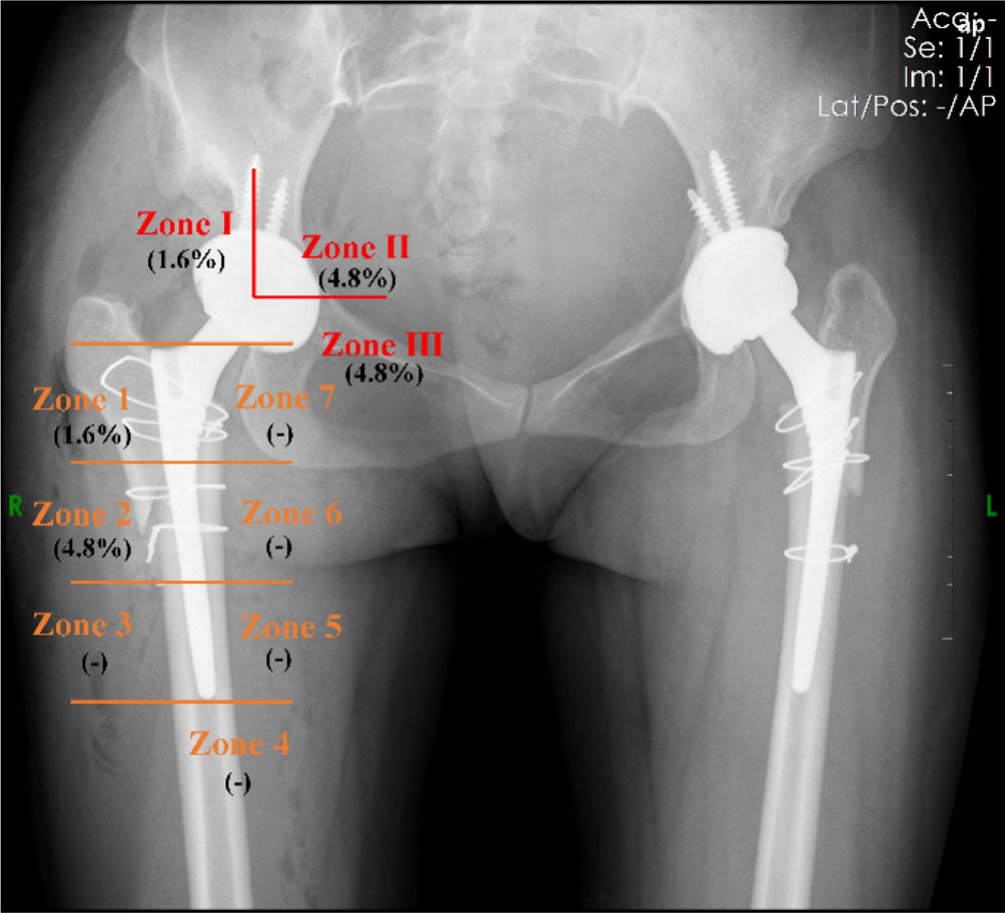
The Gruen zones and DeLee and Charnley zones are used for X‐ray analysis of periprosthetic osteolysis.

The incidence of postoperative adverse events after THA combined with proximal femoral reconstruction in this series is shown in Table 3. Out of the included 63 hips, three cases of DVT were identified, all of which were muscular vein thrombosis. Acetabular osteolysis was detected in three hips during follow‐up, mainly concentrated in zones II and III. Femoral osteolysis was found in four hips, primarily located in Gruen zone 2 (Figure 3). There was one case of nonunion at the osteotomy site and two cases of postoperative dislocation in this series. One patient suffered a nonunion of the osteotomy, possibly due to less overlap of the osteotomy ends. During follow‐up, there were no signs of proximal femoral displacement and no related clinical symptoms, so no treatment was taken. Two patients experienced dislocation after surgery, and X‐ray examination showed that inclination and anteversion were both good. One dislocation occurred due to excessive squatting, but it did not recur after we performed closed reduction and immobilization. Another case occurred under external impact, and we performed the revision by increasing the length of the femoral neck and achieved good results. There were no cases of intraoperative femoral cortical fractures or postoperative infection.

**TABLE 3:** Incidence of adverse events.

Adverse events	n (%)
Deep vein thrombosis	3 (4.8%)
Osteolysis	
Acetabular component	3 (4.8%)
Femoral stem	4 (6.4%)
Nonunion of the osteotomy	1 (1.6%)
Dislocation	2 (3.2%)
Intraoperative femoral fracture	0 (0%)
Infection	0 (0%)
Total	13 (20.6%)

## Discussion

### Principal Findings

The principal findings of this study were that THA combined with proximal femoral reconstruction shows promising and reliable clinical and radiological results for the treatment of severe hip joint deformities. This series included a total of 43 patients (63 hips) with severe hip joint deformities who were treated using THA combined with proximal femoral reconstruction technique. To the best of our knowledge, this represents a significant addition to the current body of evidence showing the utility of this technique with the largest sample size. Our results indicate that this new technique can meet the needs of complex cases, such as severe hip deformities, with a lower incidence of adverse events and lower hospitalization costs. This new method is particularly suitable for cases of high dislocation of DDH and those with extremely narrow femoral medullary canals.

### History and Comparison of Proximal Femoral Osteotomy

Historically, femoral management of hip deformities such as high dislocation DDH has been challenging for orthopedic surgeons, as it is often difficult to excavate the medullary cavity to implant the conventional‐sized femoral stem. In 1932, Ombreddanne proposed subtrochanteric shortening osteotomy, and Sponseller and McBeath *et al*.^17^ described femoral shortening osteotomy for joint replacement procedures in DDH patients. With the continuous development of this technique, several improvements and modifications have been reported, such as transverse osteotomy,^18^ oblique osteotomy,^19^ and V‐shaped osteotomy,^20^ all of which demonstrated favorable clinical outcomes and patient satisfaction. However, the complete transection of the femur below the trochanters reduces the contact area between the two sections, compromising rotational stability. In the 1960s, Charnley *et al*.^21^ proposed greater trochanter osteotomy and extended this method to joint replacement surgery, suggesting that repositioning the greater trochanter during THA can effectively increase the abductor lever arm, marking the beginning of the greater trochanter sliding osteotomy. In 1987, Glassman *et al*.^22^ elucidated the benefits of this technique, which, compared to shortening osteotomy below the trochanters, allowed for flexible adjustment of osteotomy length, preserved the bone quality between trochanters, and is primarily used in hip revision surgery.

Nevertheless, the aforementioned methods have limitations, including increased surgical difficulty and duration, as well as risks of nonunion and intraoperative fractures. A meta‐analysis of 39 studies indicated that the overall probability of nonunion after THA combined with trochanteric osteotomy for treating DDH was 3.79%, with a dislocation rate of 5.88%.^23^ In our series, over a mean follow‐up of 25.6 months, the nonunion rate was 1.6% and the dislocation rate was 3.2%, suggesting comparable or superior outcomes in treating severe proximal femoral deformities. Moreover, there was no incidence of infection in our study, while in previous studies, the incidence range was 0–1.34%.^24^, ^25^, ^26^ This might be attributed to the fact that THA combined with proximal femoral reconstruction simplifies the procedure and shortens the operating time for complex cases (mean 125.1 min), thereby reducing the incidence of postoperative infection.^27^

### Clinical Functional Outcomes and Complications

Our method provides an innovative and reliable solution for patients with severely narrow femoral medullary canals. This technique, which involves artificially remodeling the narrow canal and reconstructing it into a larger one, can mitigate the risk of intraoperative femoral cortical fractures. This is evidenced by our results, which showed no incidence of fractures, while in previous studies, the incidence ranged from 5.3% to 14.3%.^18^, ^28^, ^29^ Additionally, this procedure enables the use of conventional prostheses, eliminating the need for specialized modular prostheses such as the S‐ROM (Deputy), thus substantially reducing the cost of treatment. Additionally, our study analyzed the distribution of osteolysis, which was primarily concentrated in Gruen zone 2, the distal end of the greater trochanter bone flap. This warrants our attention and may be due to the distal end being non‐weight‐bearing due to the downward movement of the greater trochanter bone flap. However, in our follow‐up, we found no instances where this led to stem loosening.

Furthermore, our study shows that this method enhances hip function scores and reduces LLD. The improvement in HHS in this series improved from an average of 44.8 preoperatively to 89.6 postoperatively, which was like that reported in previous studies.^30^, ^31^ Additionally, the average LLD was significantly reduced from 4.1 cm to 1.3 cm, underscoring the benefits of this technique in managing leg length.

### Indications, Highlights, and Prospects of Clinical Application

THA combined with proximal femoral reconstruction techniques is primarily indicated for patients with high dislocation of DDH, sequelae of septic hip arthritis, and other severe proximal femoral deformities. The advantage of this method lies in the artificial reconstruction of the narrow femoral medullary cavity, reducing the risk of intraoperative femoral cortical fractures. Additionally, it allows for the use of traditional prostheses, avoiding the increased treatment costs associated with specialized modular prostheses. In our experience, precise osteotomy is crucial for successful surgery. To prevent iatrogenic fractures in patients with abnormal stenosis of the femoral medullary canal, especially subtrochanteric stenosis, steel wires can be pre‐bundled and fixed before inserting the press‐fit prosthesis. Meanwhile, the greater trochanteric bone flap can more easily adjust offset and abductor strength, effectively correcting limb length discrepancy. Furthermore, to reduce the risk of nonunion at the osteotomy site, we recommend resecting a larger bone flap to achieve greater bone contact. This study demonstrates that THA combined with proximal femoral reconstruction yields favorable clinical and radiological outcomes, addressing the clinical challenge of complex proximal femoral deformities. However, potential complications should be noted.

### Limitations

Some limitations of this study should be acknowledged. First, this was a retrospective study without a control group, potentially limiting our ability to interpret the results. Second, the sample size was moderate, and although it is the largest study of THA combined with proximal femoral reconstruction techniques to date, further study with a larger sample size and longer follow‐up will provide more robust evidence. Finally, this was a single‐center study and the surgical approach was determined by the surgeon's preference, which could influence the study results.

## Conclusion

The combination of THA and proximal femoral reconstruction was found to be effective in terms of clinical and radiological outcomes, with minimal complications. This innovative surgical strategy simplifies femoral shortening osteotomy, artificially expands the medullary canal, and minimizes the need for specialized prostheses. We recommend its application in complex primary THA cases involving severe proximal femoral deformities.

## Conflict of Interest Statement

We declare that we have no conflict of interest.

## Ethics Statement

Institutional ethical board review (2023‐RE‐358) was obtained for the present study. Written informed consent was obtained from the patients for the publication of this retrospective cohort study.

## Author Contributions

Concept and design: XZZ, WH and CZ. Acquisition, analysis, or interpretation of data: XYS, RWZ and JWM. Drafting of the manuscript: XYS and XZZ. Critical revision of the manuscript for important intellectual content: XZZ, WH and CZ. Statistical analysis: XYS, RWZ and JWM. Supervision: WH and CZ.

## Supporting information


**Table S1.** Details of included study cases.

## Data Availability

Anonymized data of the study can be made available from the corresponding author upon reasonable request.
