# Supplementary material for: Total Hip Arthroplasty Combined with Proximal Femoral Reconstruction Effectively Treats Severe Hip Deformities: A Novel Osteotomy Technique
Source: Orthop Surg. 2024 Jun 18;16(8):1939–45. doi: 10.1111/os.14136 (PMC11293906; doi:10.1111/os.14136)
Supplement: Supplementary file 1 — Table S1. Details of included study cases. [file OS-16-1939-s001.docx]

Table S1. Details of included study cases.

| Case | Sex | Age  (year) | Diagnosis | Operation time (mins) | Length of stay (days) | Acetabular size (mm) | Stem size | Adverse events | | | | |
| --- | --- | --- | --- | --- | --- | --- | --- | --- | --- | --- | --- | --- |
|  |  |  |  |  |  |  |  | Osteolysis | DVT | Dislocation | Nonunion | Fracture |
| 1 | M | 58 | Pyogenic hip arthritis | 150 | 12 | 52 | 15# | - | - | - | - | - |
| 2 | F | 24 | DDH (Crowe IV) | 130 | 10 | 44 | 13# | - | - | - | - | - |
|  |  |  | DDH (Crowe IV) | 135 | 11 | 44 | 14# | - | - | - | - | - |
| 3 | F | 62 | DDH (Crowe IV) | 90 | 6 | 44 | 10 | - | - | - | - | - |
|  |  |  | DDH (Crowe IV) | 80 | 5 | 46 | 9 | - | - | - |  | - |
| 4 | F | 59 | Secondary deformity | 70 | 6 | 48 | 15# | - | - | - | - | - |
| 5 | F | 38 | Secondary deformity | 90 | 10 | 48 | 9 | Stem: zone 6 | - | - | - | - |
|  |  |  | DDH (Crowe IV) | 95 | 5 | 48 | 11 | - | - | - |  | - |
| 6 | F | 51 | DDH (Crowe IV) | 57 | 7 | 46 | 9 | Acetabular loosening  Stem: zone 6 | - | - | - | - |
| 7 | F | 40 | DDH (Crowe III) | 90 | 8 | 46 | 8 | - | - | - | - | - |
|  |  |  | DDH (Crowe IV) | 125 | 6 | 42 | 13# | - | - | - | - | - |
| 8 | F | 33 | DDH (Crowe IV) | 180 | 11 | 44 | 13# | - | DVT | - | - | - |
| 9 | M | 63 | DDH (Crowe IV) | 225 | 10 | 54 | 9 | - | DVT | Dislocation | - |  |
| 10 | F | 29 | DDH (Crowe IV) | 153 | 8 | 44 | 8 | - | - | - | - | - |
|  |  |  | DDH (Crowe IV) | 120 | 6 | 44 | 8 | - | - | - | - | - |
| 11 | F | 37 | DDH (Crowe IV) | 120 | 7 | 44 | 8 | - | - | - | - | - |
|  |  |  | DDH (Crowe IV) | 140 | 8 | 44 | 8 | - | - | - | - | - |
| 12 | F | 35 | DDH (Crowe II) | 90 | 7 | 44 | 8 | Stem: zone 6 | - | - | - |  |
| 13 | F | 45 | DDH (Crowe IV) | 50 | 10 | 48 | 9 | - | - | - | - | - |
| 14 | F | 45 | Secondary deformity | 80 | 10 | 44 | 8 | - | - | - | - |  |
| 15 | F | 58 | Pyogenic hip arthritis | 85 | 7 | 46 | 9 | - | - | - | - | - |
| 16 | F | 25 | DDH (Crowe IV) | 120 | 31 | 44 | 6 | - | - | - | - | - |
|  |  |  | DDH (Crowe IV) | 95 | 9 | 44 | 8 | - | - | Dislocation | - | - |
| 17 | F | 33 | DDH (Crowe IV) | 145 | 9 | 44 | 13# | - | - | - | - | - |
| 18 | F | 41 | DDH (Crowe IV) | 125 | 7 | 46 | 6 | - | - | - | - | - |
|  |  |  | DDH (Crowe IV) | 270 | 12 | 52 | 6 | - | - | - | - | - |
| 19 | F | 26 | DDH (Crowe IV) | 115 | 8 | 44 | 8 | - | - | - | - | - |
|  |  |  | DDH (Crowe IV) | 105 | 12 | 44 | 8 | - | - | - | - | - |
| 20 | M | 27 | DDH (Crowe IV) | 101 | 7 | 44 | 9 | - | - | - | - | - |
|  |  |  | DDH (Crowe IV) | 105 | 7 | 44 | 9 | - | - | - | - | - |
| 21 | F | 23 | DDH (Crowe IV) | 125 | 7 | 44 | 8 | Stem: zone 6 | - | - | - | - |
| 22 | F | 22 | DDH (Crowe IV) | 100 | 11 | 44 | 6 | - | - | - | - | - |
|  |  |  | DDH (Crowe IV) | 105 | 9 | 44 | 6 | - | - | - | - | - |
| 23 | F | 59 | DDH (Crowe IV) | 110 | 10 | 46 | 8 | - | DVT | - | - | - |
| 24 | F | 31 | DDH (Crowe IV) | 105 | 10 | 44 | 6 | - | - | - | - | - |
|  |  |  | DDH (Crowe IV) | 125 | 7 | 44 | 6 | - | - | - | - | - |
| 25 | M | 17 | DDH (Crowe IV) | 130 | 6 | 46 | 9 | - | - | - | - | - |
| 26 | F | 48 | DDH (Crowe IV) | 160 | 9 | 44 | 8 | - | - | - | - | - |
| 27 | M | 51 | DDH (Crowe IV) | 177 | 11 | 48 | 8 | - | - | - | - | - |
|  |  |  | DDH (Crowe IV) | 145 | 9 | 44 | 6 | - | - | - | - | - |
| 28 | F | 52 | DDH (Crowe IV) | 137 | 9 | 38 | 6 | - | - | Dislocation | - | - |
|  |  |  | DDH (Crowe IV) | 145 | 5 | 44 | 8 | - | - | - | - | - |
| 29 | M | 30 | DDH (Crowe IV) | 176 | 7 | 50 | 10 | - | - | - | - | - |
| 30 | F | 35 | DDH (Crowe IV) | 193 | 9 | 44 | 6 | - | - | Dislocation | - | - |
| 31 | M | 35 | Secondary deformity | 105 | 8 | 54 | 9 | Acetabular: zone II and III | - | - | - | Fracture |
| 32 | F | 38 | DDH (Crowe III) | 96 | 5 | 44 | 6 | - | - | - | - | - |
|  |  |  | DDH (Crowe III) | 104 | 6 | 44 | 6 | - | - | - | - | - |
| 33 | F | 52 | DDH (Crowe IV) | 105 | 6 | 44 | 8 | - | - | - | - | - |
|  |  |  | DDH (Crowe IV) | 187 | 10 | 44 | 6 | - | - | - | - | - |
| 34 | F | 35 | DDH (Crowe IV) | 125 | 8 | 44 | 6 | - | - | - | - | - |
|  |  |  | DDH (Crowe IV) | 150 | 7 | 44 | 6 | - | - | - | - | - |
| 35 | F | 57 | DDH (Crowe IV) | 125 | 8 | 44 | 8 | - | - | - | - | - |
| 36 | F | 38 | DDH (Crowe IV) | 110 | 6 | 44 | 6 | - | - | - | - | - |
|  |  |  | DDH (Crowe IV) | 125 | 5 | 44 | 6 | - | - | - | - | - |
| 37 | F | 31 | DDH (Crowe IV) | 185 | 9 | 44 | 8 | - | - | - | - | - |
|  |  |  | DDH (Crowe IV) | 190 | 7 | 44 | 8 | - | - | - | - | - |
| 38 | F | 50 | DDH (Crowe IV) | 126 | 12 | 44 | 6 | - | - | - | - | - |
| 39 | F | 50 | DDH (Crowe IV) | 95 | 5 | 48 | 8 | - | - | - | - | - |
| 40 | M | 27 | Juvenile idiopathic osteoarthritis | 100 | 10 | 44 | 6 | - | - | - | - | - |
| 41 | F | 33 | DDH (Crowe IV) | 110 | 10 | 44 | 6 | - | - | - | - | - |
|  |  |  | DDH (Crowe IV) | 170 | 8 | 48 | 8 | - | - | - | - | - |
| 42 | F | 35 | DDH (Crowe IV) | 100 | 8 | 44 | 6 | - | - | - | - | - |
| 43 | F | 24 | DDH (Crowe IV) | 105 | 7 | 44 | 6 | - | - | - | - | - |

Note: DDH, Developmental dysplasia of the hip; DVT, Deep vein thrombosis. #Represent the case used Wagner Cone (Zimmer, Warsaw, USA) stem, others used Corail Hip System (DePuy Synthes, USA).
